# Necroptosis promotes cell-autonomous activation of proinflammatory cytokine gene expression

**DOI:** 10.1038/s41419-018-0524-y

**Published:** 2018-04-27

**Authors:** Kezhou Zhu, Wei Liang, Zaijun Ma, Daichao Xu, Shuangyi Cao, Xiaojuan Lu, Nan Liu, Bing Shan, Lihui Qian, Junying Yuan

**Affiliations:** 1 Interdisciplinary Research Center on Biology and Chemistry, Shanghai Institute of Organic Chemistry, Chinese Academy of Sciences, 26 Qiuyue Rd, PuDong District, 201203 Shanghai, China; 2 University of Chinese Academy of Sciences, 100049 Beijing, China; 3 Department of Cell Biology, Harvard Medical School, 240 Longwood Avenue, Boston, MA 02115 USA

## Abstract

Necroptosis, a form of regulated necrotic cell death, is mediated by receptor interacting protein 1 (RIPK1), RIPK3, and mixed lineage kinase domain-like protein (MLKL). However, the mechanism by which necroptosis promotes inflammation is still unclear. Here we report that the expression of cytokines is robustly upregulated in a cell-autonomous manner during necroptosis induced by tumor necrosis factor alpha (TNFα). We demonstrate that TNFα-induced necroptosis leads to two waves of cytokine production. The first wave, more transient and weaker than the second, is in response to TNFα alone; whereas the second wave depends upon the necroptotic signaling. We show that necroptosis promotes the transcription of TNFα-target genes in a cell-intrinsic manner. The activation of both NF-κB and p38 by the necroptotic machinery, RIPK1, RIPK3, and MLKL, is involved in mediating the robust induction of cytokine expression in the second wave. In contrast, necroptosis induced by direct oligomerization of MLKL promotes cytokine production at much lower levels than that of necroptosis induced with TNFα. Thus, we conclude that TNFα-induced necroptosis signaling events mediated by RIPK1 and RIPK3 activation, in addition to the MLKL oligomerization, promotes the expression of cytokines involving multiple intracellular signaling mechanisms including NF-κB pathway and p38. These findings reveal that the necroptotic cell death machinery mounts an immune response by promoting cell-autonomous production of cytokines. Our study provides insights into the mechanism by which necroptosis promotes inflammation in human diseases.

## Introduction

Necroptosis is a regulated form of necrotic cell death that can be activated when cells are stimulated by the proinflammatory cytokine tumor necrosis factor alpha (TNFα) under apoptosis-deficient conditions^1,2^. While necrosis is known to promote inflammation by the passive release of the damage-associated molecular pattern molecules (DAMPs) from ruptured cell membrane, the mechanism by which necroptosis promotes inflammation has not been vigorously examined. In TNFα-stimulated cells, necroptosis is activated via the formation of two sequential complexes, complex I and complex IIb. Receptor interacting protein 1 (RIPK1) is recruited into complex I by interacting with the intracellular death domain of TNF receptor 1 (TNFR1). Inhibition of apoptosis promotes the activation of RIPK1. Activated RIPK1 interacts with RIPK3 to induce its phosphorylation and formation of the RIPK1/RIPK3 complex, known as complex IIb^3,4^. Activated RIPK3 further recruits and phosphorylates the pseudokinase mixed lineage kinase domain-like protein (MLKL). Phosphorylated MLKL in turn oligomerizes and translocates from the cytosol to the plasma membrane to execute cell death^5–7^.

TNFα promotes inflammation via nuclear factor κB (NF-κB) -regulated transcriptional program^8^. Under basal conditions, NF-κB, a dimeric transcription factor complex including the Rel family of proteins, is sequestered in the cytoplasm by inhibitor of NF-κB (IκB). RIPK1 acts as a scaffold to activate NF-κB^9–11^. The recruitment and ubiquitination of RIPK1 in the TNFα receptor signaling complex promotes the activation of TGF-β-activated kinase 1 (TAK1), which in turn phosphorylates and activates IκB kinase (IKK) complex^12,13^. Activated IKKs then phosphorylate IκB to promote its ubiquitination by SCF-β-TrCP and subsequent degradation through the proteasomal pathway, thereby allowing the NF-κB complex to translocate into the nucleus to activate transcription^14–16^.

Here, we investigate the mechanism by which necroptosis promotes inflammation. We show that TNFα-induced necroptosis signaling events involving RIPK1 and RIPK3 activation, in addition to the MLKL oligomerization, promote the expression of proinflammatory cytokines cell-autonomously through intracellular signaling mechanisms including NF-κB pathway and p38.

## Results

### Upregulation of cytokines during necroptosis

**Fig. 1:**
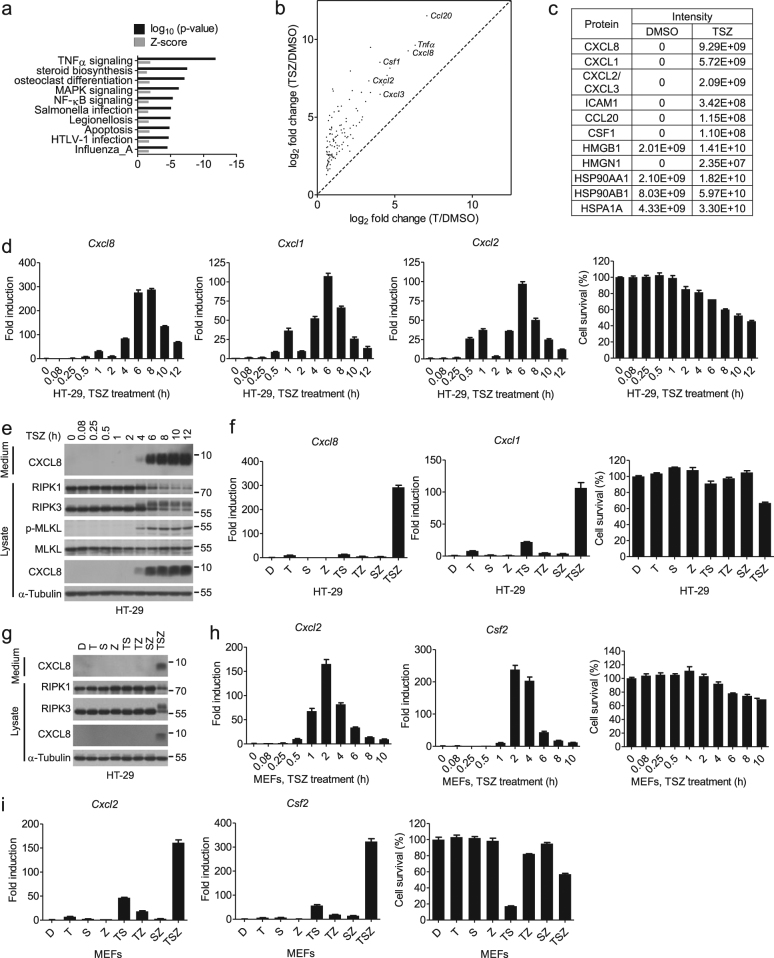
Expression of cytokines is highly induced during necroptosis. **a** The Kyoto Encyclopedia of Genes and Genomes (KEGG) pathway database analysis of the 813 necroptosis-upregulated genes in Enrichr. **b** Scatterplot of the fold inductions of the overlapping genes induced by TNFα and TSZ. Representative genes were labeled. **c** Table of selected proteins identified in the media of necroptotic cells by mass spectrometry. Protein abundance was quantified with LFQ module implemented in MaxQuant. **d** HT-29 cells were treated with TSZ for the indicated periods of time. *Cxcl8*,* Cxcl1*, and *Cxcl2* mRNA levels were measured by qPCR. The cell viability was determined by CellTiter-Glo. **e** HT-29 cells were treated with TSZ for the indicated periods of time. The cell lysates and culture media were collected separately, and analyzed by western blotting with indicated antibodies. **f** HT-29 cells were treated as indicated for 8 h. The expression levels of *Cxcl8* and *Cxcl1* were analyzed by qPCR. The cell viability was determined by CellTiter-Glo. D, DMSO (<0.2%). **g** HT-29 cells were treated as indicated for 8 h. The supernatants and cell lysates were collected and analyzed by western blotting. **h** MEFs were treated for the indicated periods of time with TSZ. The expression levels of *Cxcl2 and Csf2* were determined by qPCR. The cell viability was determined by CellTiter-Glo. **i** MEFs were treated as indicated. *Cxcl2* and *Csf2* mRNA levels were measured by qPCR after 4 h of treatment. The cell viability was determined by CellTiter-Glo after 13 h of treatment. Gene expression determined by qPCR was shown as fold induction compared with untreated cells in all figures. All reagents were used at concentrations as described in Materials and Methods in all experiments, unless otherwise noted. Data were presented as mean ± SEM of triplicates

To characterize the transcriptional changes in necroptotic cells, we stimulated HT-29 cells with TNFα (T), SM-164 (S), and a pan-caspase inhibitor zVAD (Z) (TSZ), a well-established protocol to induce TNFα-mediated necroptosis, and profiled the transcriptome of necroptotic cells by RNA-sequencing (RNA-seq). Based on the differential gene expression analysis, we identified a transcriptional signature of necroptosis consisting of 813 genes whose expression was upregulated >1.5 fold (*n* = 3, *p* < 0.01) by TSZ and inhibited >2 fold (*n* = 3, *p* < 0.01) by the addition of Nec-1s, a chemical inhibitor of necroptosis against the RIPK1 kinase^17,18^. The most-significantly enriched gene set during necroptosis was associated with the TNFα signaling (Fig. 1a)^19,20^. Consistently, the expression of 116 out of the 813 genes activated by necroptosis was also induced by TNFα alone (*n* = 3, T/DMSO > 1.5, *p* < 0.05), but at a much lower level than that by TSZ (Fig. 1b). The mRNA levels of proinflammatory cytokines, such as *Ccl20*, *Tnfα*, *Cxcl8*, and *Csf1*, well-known TNFα target genes, were the most highly upregulated during necroptosis (Fig. 1b).

We further analyzed the proteins/cytokines released from necroptotic cells using mass spectrometry. Apart from the released intracellular proteins such as high mobility group (HMG) proteins, including HMGB1 and HMGN1^21,22^, the induction of necroptosis was associated with increased release of cytokines, such as CXCL8, CXCL1, CCL20, and CSF1, in the culture media (Fig. 1c).

We next characterized the temporal profiles of representative cytokine expression by quantitative PCR (qPCR). We found that there were two waves of increases in the mRNA levels of *Cxcl8*, *Cxcl1*, and *Cxcl2* (Fig. 1d). The first wave increases occurred around 1 h in HT-29 cells after the addition of TSZ and declined rapidly. The second wave occurred when the cells began to die and corresponded temporally with the phosphorylation of RIPK3 and MLKL (Fig. 1d, e). Compared to that of the first wave, the increased expression of cytokines in the second wave was much more robust and persistent. In contrast to that of necroptotic cells induced by TSZ, the induction of cytokines, such as *Cxcl8* and *Cxcl1*, was much weaker or not induced at all in cells treated with TNFα alone or SM-164 alone, or in apoptotic cells induced with TNFα/SM-164 (TS) (Fig. 1f, g). The time course analysis of cytokine expression stimulated by TNFα and TSZ showed that although a comparable level of cytokine expression was induced by TNFα and TSZ at 1 h following the treatment, the second wave of cytokine induction in necroptosis was absent in cells stimulated by TNFα alone (Supplementary Figure 1a). Thus, necroptosis enhanced cytokine production by promoting a second wave of cytokine expression that cannot be triggered by TNFα alone.

To determine if necroptosis is generally associated with increased production of cytokines, we stimulated other cell lines with TSZ to activate necroptosis. We found that the expression of cytokines was induced during necroptosis in mouse embryonic fibroblasts (MEFs), wild-type Jurkat, HT-22, and L929 cells, which synchronized with the progress of cell death (Fig. 1h and Supplementary Figure 1b–e). Similarly, the robust induction of *Cxcl2* and *Csf2* was specific to necroptotic cells induced by TSZ as minimal levels of cytokines were detected in MEFs stimulated by other treatments (Fig. 1i and Supplementary Figure 1f). In MEFs, the second wave of cytokine expression induced by TSZ followed immediately the first one (Supplementary Figure 1f).

We further verified that cytokine production could be induced in necroptosis triggered by stimuli other than TSZ. We found that cytokine expression could be induced during necroptosis of FADD-deficient Jurkat cells with TNFα alone, as well as HT-29 cells and wild-type Jurkat cells with a combination of TRAIL, SM-164, and zVAD (Supplementary Figure 1g–i). Taken together, these results suggest that the expression of proinflammatory cytokines can be induced in multiple necroptosis models.

### NF-κB is required for cytokine induction during necroptosis

**Fig. 2:**
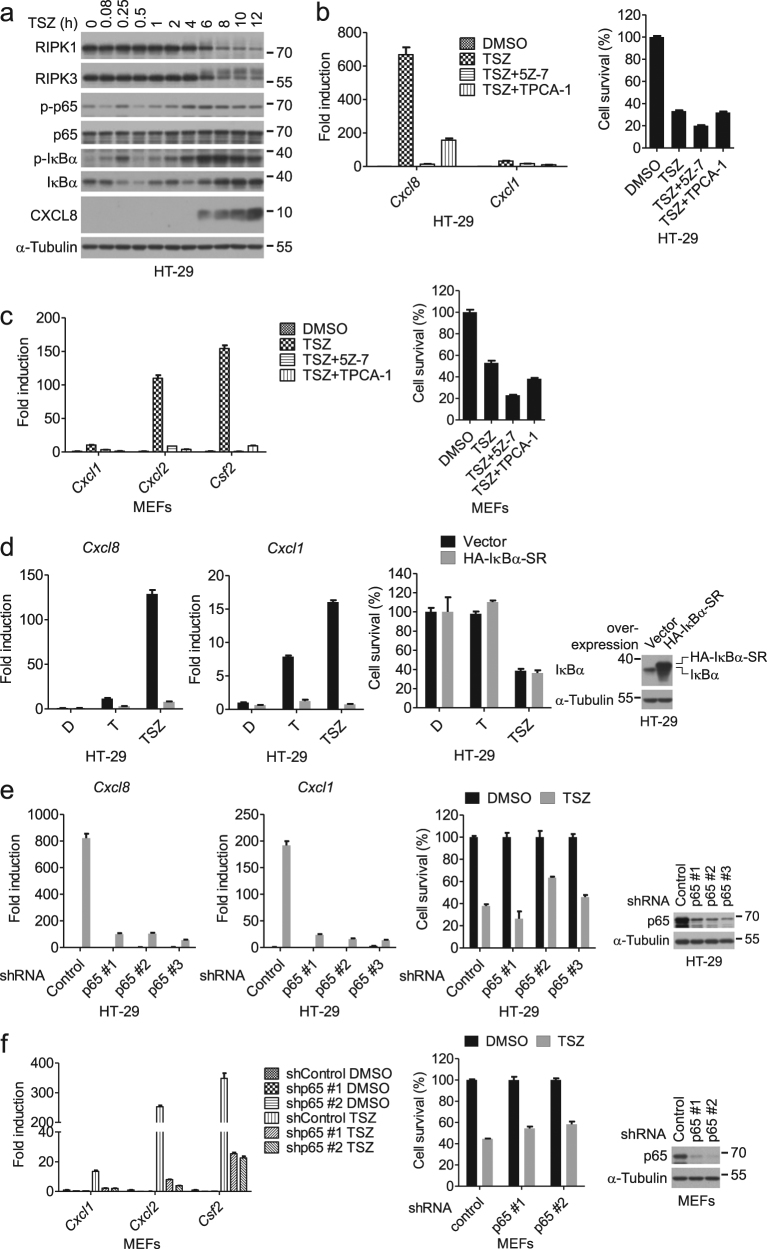
NF-κB regulates the cytokine induction during necroptosis. **a** HT-29 cells were treated with TSZ for the indicated periods of time. The cell lysates were collected for western blotting with indicated antibodies. **b** HT-29 cells were treated as indicated. *Cxcl8* and *Cxcl1* mRNA levels were measured by qPCR after 8 h of treatment (left). The cell viability was determined by CellTiter-Glo after 12 h of treatment (right). **c** MEFs were treated as indicated. The mRNA levels of *Cxcl1*, *Cxcl2*, and *Csf2* were measured by qPCR after 4 h of treatment (left). The cell viability was determined by CellTiter-Glo after 13 h of treatment (right). **d** HT-29 cells overexpressing HA-IκBα-SR or vector were treated as indicated. The expression of *Cxcl8* and *Cxcl1* was analyzed by qPCR at 8 h post treatment. The cell viability was determined by CellTiter-Glo after 24 h of treatment. The expression of IκBα was analyzed by western blotting (right panel). **e** HT-29 cells stably expressing control shRNA or shRNA targeting p65 were treated as indicated. The mRNA levels of *Cxcl8* and *Cxcl1* were measured by qPCR after 8 h of treatment. The cell viability was determined by CellTiter-Glo after 30 h of treatment. The knockdown efficiency was determined by western blotting (right panel). **f** MEFs stably expressing control shRNA or shRNA targeting p65 were treated as indicated. The mRNA levels of *Cxcl1*, *Cxcl2*, and *Csf2* were measured by qPCR after 4 h of treatment. The cell viability was determined by CellTiter-Glo after 24 h of treatment. The knockdown efficiency was determined by western blotting (right panel). Data were represented as mean ± SEM of triplicates

Since the inhibition of transcription by actinomycin D blocked the expression of cytokines induced by necroptosis (Supplementary Figure 2), we focused our analysis on the known transcriptional mechanisms. We first tested whether the NF-κB pathway activated by TNFα was involved. We found that TSZ led to two waves of phospho-p65 and phospho-IκBα, key signal transduction events for the activation of NF-κB (Fig. 2a and Supplementary Figure 3a). Furthermore, we found that the addition of TAK1 inhibitor (5Z)-7-Oxozeaenol (5Z-7) and IKK inhibitor TPCA-1 abolished the induction of the cytokines by TSZ (Fig. 2b, c, and Supplementary Figure 3b–d), suggesting that the activation of the IKK complex is required for cytokine expression during necroptosis.

To examine if the activation of IKKs is required for the second wave of cytokine production during necroptosis independent of the first wave, we delayed the addition of TAK1 and IKK inhibitors to specifically block the second wave of IKK activation and found that delayed inhibition of IKK still could block the second wave of cytokine expression and phosphorylation of p65 and IκBα (Supplementary Figure 3e–i). Furthermore, blocking NF-κB activation by either overexpression of the IκBα super-repressor (IκBα-SR) with the S32A/S36A double mutation, and therefore resistant to degradation^23^, or knockdown of p65 by short hairpin RNA (shRNA) completely abrogated cytokine expression in HT-29 cells and MEFs treated with TSZ without effects on cell death (Fig. 2d–f). Collectively, these data suggest that NF-κB is required for the second wave of cytokine expression during necroptosis.

To examine whether there are additional mechanisms underlying the cytokine induction during necroptosis, we evaluated the potential roles of MAPKs, such as p38 and JNK activated during necroptosis (Supplementary Figure 3a)^24^, and the noncanonical NF-κB pathway. The JNK inhibitor SP600125 did not inhibit the cytokine induction during necroptosis (Supplementary Figure 4a). Inhibition of p38 activity by PH-797804 or silencing p38α, the most abundant p38 member, resulted in a moderate reduction of cytokine expression by 20–50% (Supplementary Figure 4a, b). Smac mimetics had been reported to activate the noncanonical NF-κB pathway^25,26^. However, knockdown of RELB, a key mediator of noncanonical NF-κB pathway, not only did not block chemokine expression, but rather promoted *Cxcl1* expression to some extent (Supplementary Figure 4c). These results indicate that the activation of both canonical NF-κB and p38 is involved in mediating cytokine induction during necroptosis.

### The RIPK1–RIPK3–MLKL axis is essential for the induction of cytokines during necroptosis

**Fig. 3:**
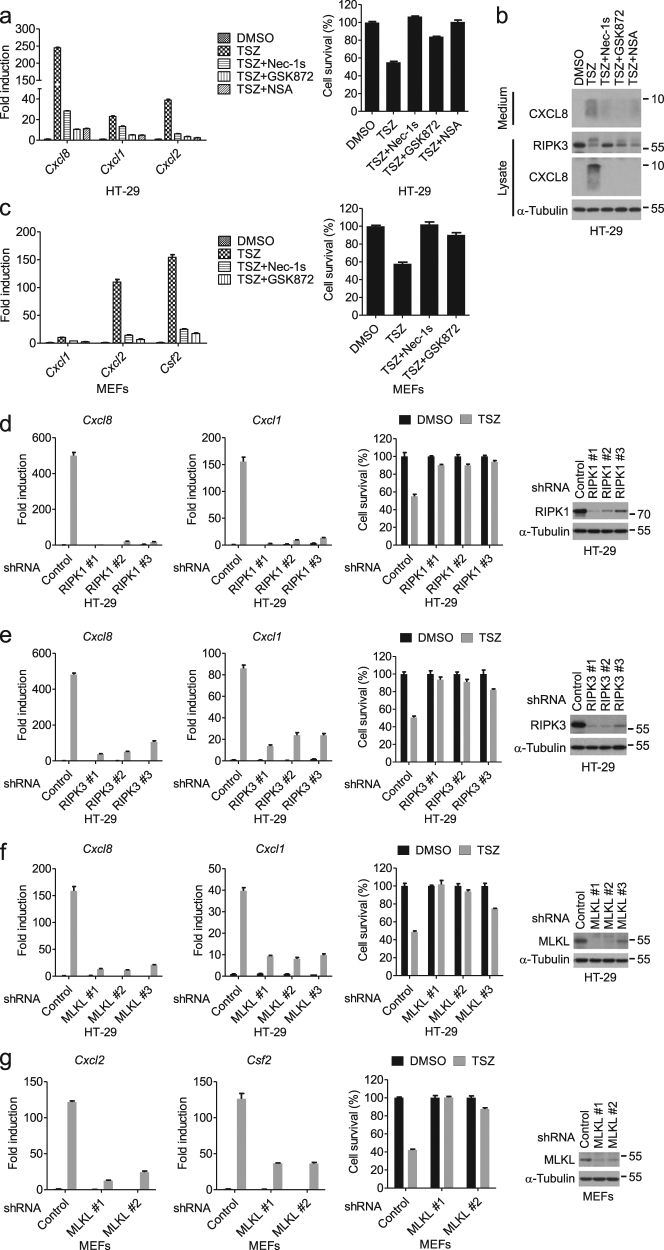
The RIP1–RIP3–MLKL axis is required for the induction of cytokines associated with necroptosis. **a** HT-29 cells were treated as indicated. The mRNA levels of *Cxcl8* and *Cxcl1* were measured by qPCR after 8 h of treatment (left). The cell viability was determined by CellTiter-Glo after 24 h of treatment (right). **b** HT-29 cells were treated as indicated for 8 h. Cell culture medium and cell lysates were collected separately, followed by western blotting analysis. **c** MEFs were treated as indicated. The mRNA levels of *Cxcl1*, *Cxcl2*, and *Csf2* were measured by qPCR after 4 h of treatment (left). Cell viability was determined by measuring ATP levels after 13 h of treatment (right). **d**–**f** HT-29 cells stably expressing the indicated shRNA were treated with DMSO or TSZ. The mRNA levels of *Cxcl8* and *Cxcl1* were measured by qPCR after 8 h of treatment. The cell viability was determined by CellTiter-Glo after 24 h of treatment. The knockdown efficiency was determined by western blotting. **g** MEFs stably expressing the indicated shRNA were treated with DMSO or TSZ. The mRNA levels of *Cxcl2* and *Csf2* were measured by qPCR after 4 h of treatment. The cell viability was determined by CellTiter-Glo after 22 h of treatment. The knockdown efficiency was determined by western blotting. Data were represented as mean ± SEM of triplicates

We next examined the involvement of necroptotic machinery in the production of cytokines. Interestingly, we found that cytokine inductions by necroptosis were inhibited by Nec-1s (an inhibitor of RIPK1), GSK872 (an inhibitor of RIPK3), and necrosulfonamide (NSA, an inhibitor of human MLKL oligomerization) (Fig. 3a–c and Supplementary Figure 1g–i, 5a–d)^17,27,28^. In addition, knockdown of RIPK1, RIPK3, and MLKL reduced the production of cytokines (Fig. 3d–g and Supplementary Figure 5e–h). On the other hand, inhibition of RIPK1, RIPK3, or MLKL failed to block the first peak of *Cxcl8* expression induced by TSZ, or the expression of cytokine induced by TNFα alone or TS in HT-29 cells (Supplementary Figure 6a–d). Also, MLKL ablation did not affect the induction of *Cxcl8* and *Cxcl1* by TNFα alone or TS (Supplementary Figure 6e). Thus, the RIPK1–RIPK3–MLKL axis is specifically required for the necroptosis-induced cytokine production.

### Cell-autonomous induction of cytokines during necroptosis

**Fig. 4:**
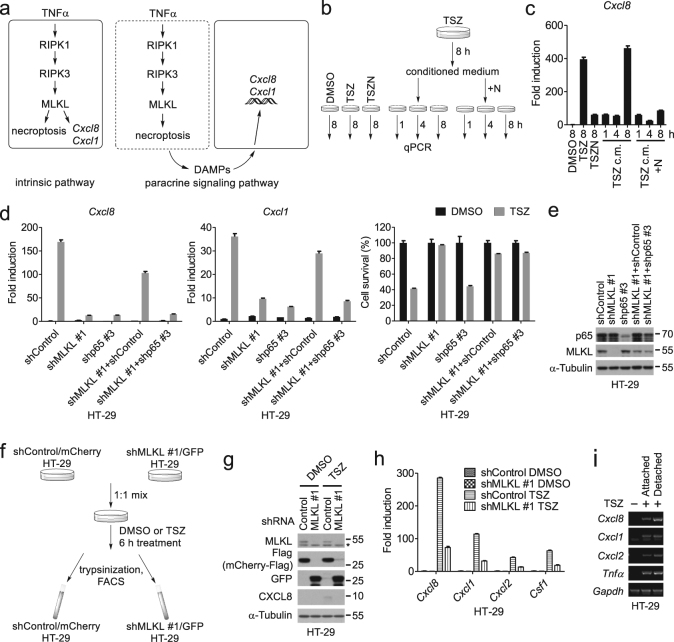
Cytokine production during necroptosis is mediated by a cell intrinsic pathway. **a** The cell-intrinsic and paracrine signaling hypothesis. **b** Schematic of the treatments for HT-29 cells with indicated compound(s) or conditioned medium. N, 10 μM Nec-1s. **c** HT-29 cells were treated as shown in **b**. The expression of *Cxcl8* was determined by qPCR; c.m., conditioned medium. **d**, **e** HT-29 cells expressing MLKL shRNA or p65 shRNA were cultured individually or co-cultured in a ratio 1:1, and stimulated with DMSO or TSZ. The mRNA levels of *Cxcl8* and *Cxcl1* were measured by qPCR after 8 h of treatment and the cell viability was determined by CellTiter-Glo after 29 h of treatment (**d**). The protein levels of p65 and MLKL were analyzed by western blotting (**e**). **f** Schematic of the co-culture and FACS experiment. HT-29 cells were treated with DMSO or TSZ for 6 h, and then separated by FACS to obtain Cherry+ (shControl) or GFP+ (shMLKL) populations. **g** Western blotting analysis of lysates from HT-29 cells separated in **f**. Asterisk indicates nonspecific bands. **h** Quantitative PCR analysis of *Cxcl8*, *Cxcl1*, *Cxcl2*, and *Csf1* mRNA levels in HT-29 cells derived from **f**. **i** The expression of the cytokines was determined by RT-PCR in the attached or detached HT-29 cells after 8 h treatment with TSZ

We next asked whether RIPK1–RIPK3–MLKL induced cytokines in a cell-autonomous manner, or indirectly by the release of DAMPs, which might trigger cytokine expression in a paracrine manner (Fig. 4a). To address this question, we treated naive HT-29 cells with conditioned medium from necroptotic cells and compared the time course of the cytokine induction with that directly induced by TSZ (Fig. 4b). We found that the conditioned medium from TSZ-treated HT-29 cells did not accelerate cytokine production as it still took 8 h, the same amount of time as that of inducing the expression of *Cxcl8* by TSZ (Fig. 4c). In addition, the conditioned medium from necroptotic cells did not compromise the ability of Nec-1s to inhibit *Cxcl8* expression in naive cells (Fig. 4c). Thus, the released DAMPs from necroptotic cells are unlikely to be sufficient to induce the expression of cytokines.

To determine if the interaction of dying cells with neighboring cells was required for the induced expression of cytokines, we co-cultured cell death-deficient shMLKL HT-29 cells and NF-κB-deficient shp65 HT-29 cells. Most notably, after necroptosis induction, the mixed culture of shMLKL cells and shp65 cells was still unable to induce the expression of *Cxcl8* and *Cxcl1* as that of shControl cells (Fig. 4d, e), suggesting that any factors from dying shp65 cells could not complement MLKL knockdown to induce cytokine expression.

To further evaluate the role of DAMPs and cell–cell interactions in cytokine production during necroptosis, we co-cultured mCherry marked shControl and GFP marked shMLKL HT-29 cells in 1:1 ratio so that they could be treated in the same well and then separated according to their fluorescent markers by FACS for analyzing the expression of cytokines (Fig. 4f, g). Consistently, the mRNA levels of cytokines in necroptotic shControl cells were much higher than that of shMLKL cells treated with TSZ (Fig. 4h).

Finally, we assayed the cytokine production in the detached dead HT-29 cells treated with TSZ. We found that the expression of cytokines in the detached cells was higher than that of the attached cells (Fig. 4i), consistent with induction of cytokine expression in necroptotic cells. From these results, we conclude that the expression of cytokines during necroptosis is induced in a cell-autonomous manner.

### The RIPK1–RIPK3–MLKL axis is required for nuclear entry of p65

**Fig. 5:**
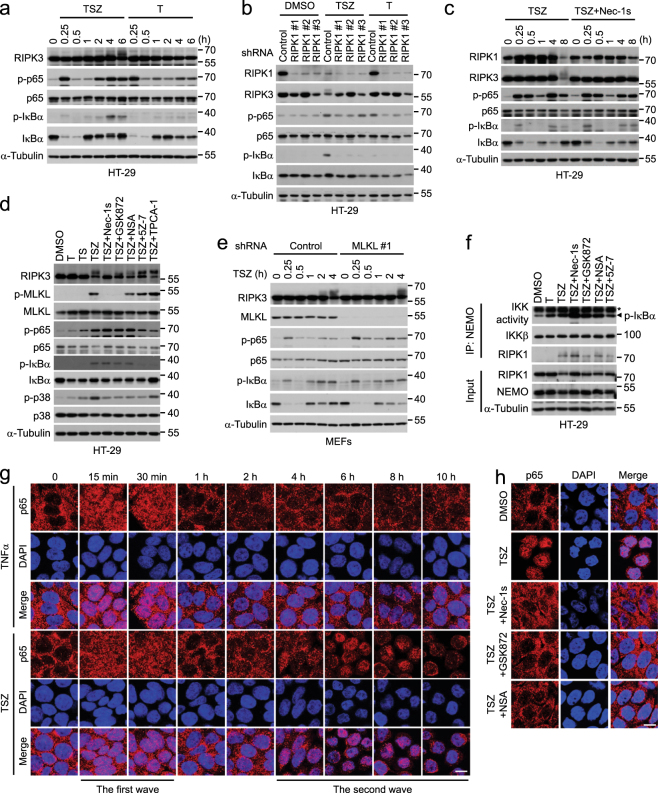
The RIP1–RIP3–MLKL axis is required for promoting p65 nuclear entry in necroptosis. **a** Western blotting analysis of lysates from HT-29 cells treated with TSZ or T for the indicated periods of time. **b** HT-29 cells stably expressing the indicated shRNAs were treated with DMSO, TSZ, or T for 8 h. The cell lysates were collected for western blotting analysis. **c** Western blotting analysis of lysates from HT-29 cells treated as indicated. **d** Western blotting analysis of lysates from HT-29 cells treated as indicated for 8 h. **e** MEFs stably expressing control or MLKL shRNA were treated as indicated. Western blotting was performed with indicated antibodies. **f** HT-29 cells were treated as indicated for 8 h. The IKK complex was immunoprecipitated with an anti-NEMO antibody and subjected to an in vitro kinase assay by probing the phosphorylated GST-IκBα. The immunoprecipitates and cell lysates were western blotted with indicated antibodies. Asterisk indicates the bands of IgG. **g** Immunostaining of p65 in HT-29 cells treated with TSZ for the indicated time periods. **h** Immunostaining of p65 in HT-29 cells treated as indicated for 8 h. Scale bars, 10 μm

We next compared the NF-κB activation between TSZ and TNFα treatment. Consistent with the magnitude of cytokine induction, the second peak of phosphorylation of p65 and IκBα during necroptosis was much stronger than that triggered by TNFα alone (Fig. 5a and Supplementary Figure 7a). In line with the requirement for the scaffolding function of RIPK1, but not its kinase activity, in activating IKK^29^, knockdown of RIPK1, but not its kinase inhibitor Nec-1s, blocked the IKK-mediated p65 and IκBα phosphorylation during necroptosis (Fig. 5b, c). Similarly, we found that neither the treatment of additional necroptosis inhibitors, including the RIPK3 inhibitor GSK872 and the MLKL inhibitor NSA, nor the silencing of RIPK3 and MLKL had any inhibitory effect on the phosphorylation of IκBα or p65 induced by TSZ (Fig. 5d, e and Supplementary Figure 7b–f). Consistent with a role of p38 in cytokine production, phosphorylation of p38, the hallmark for p38 activation, was attenuated by necroptosis inhibitors (Fig. 5d).

Because IκBα is degraded upon its phosphorylation by IKK, we further determined the kinase activity of IKK with a fusion GST-IκBα substrate using the IKK complex immunoprecipitated from the cells treated with TSZ in the presence or absence of necroptosis inhibitors. Inhibition of RIPK1, RIPK3, or MLKL could not block the IKK activity in the in vitro kinase assay (Fig. 5f). Taken together, these results suggest that the RIPK1 kinase activity, RIPK3, and MLKL enhance p38 activation and the NF-κB-dependent transcription downstream of IKK activation during necroptosis.

We next examined the nuclear translocation of p65, during necroptosis, a critical event for NF-κB. Similar to that of cytokine induction, two waves of p65 nuclear translocation occurred during TSZ-induced necroptosis (Fig. 5g). An early transient nuclear import of p65 was observed at 15 and 30 min after TSZ stimulation, and a delayed and prolonged nuclear translocation of p65 occurred at 4 h after TSZ treatment and lasted for 4 h. In contrast, only a brief nuclear accumulation was found in cells stimulated by TNFα alone at 15 and 30 min after stimulation (Fig. 5g). Thus, necroptosis is characterized with a sustained p65 nuclear translocation that is correlated temporally with the cell death and robust cytokine gene expression during the second wave. We next asked whether the RIPK1–RIPK3–MLKL axis was required for the necroptosis-specific nuclear entry of p65. We found that the inhibition of RIPK1 kinase by Nec-1s, RIPK3 by GSK872, or MLKL by NSA prevented the translocation of p65 into the nucleus (Fig. 5h). Thus, necroptotic machinery promotes the sustained nuclear entry of p65 to activate the cytokine expression during necroptosis.

### Degradation of IκBα is regulated by the RIPK1–RIPK3–MLKL axis during necroptosis

**Fig. 6:**
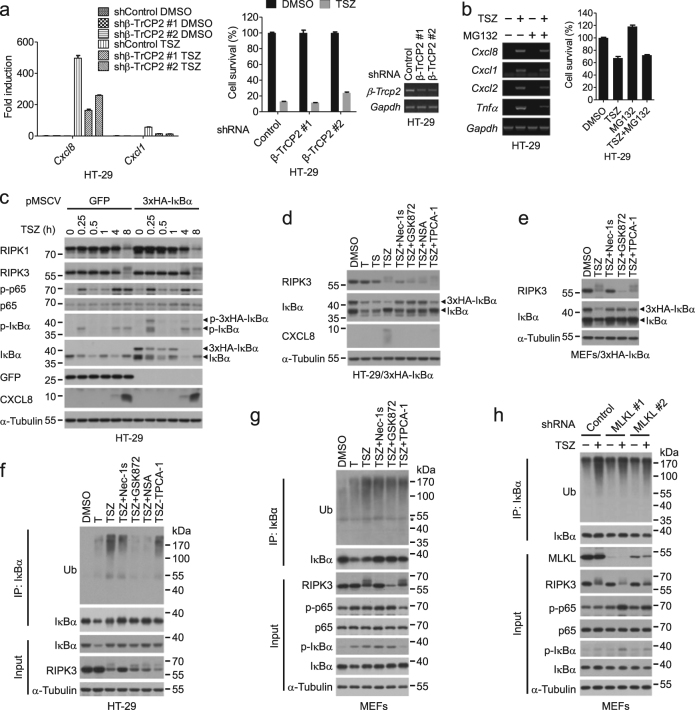
Degradation of IκBα is promoted by the RIPK1–RIPK3–MLKL axis during necroptosis. **a** HT-29 cells stably expressing control shRNA or shRNA targeting β-TrCP2 were treated as indicated. The mRNA levels of *Cxcl8* and *Cxcl1* were measured by qPCR after 8 h of treatment. The cell viability was determined by CellTiter-Glo after 32 h of treatment. The knockdown efficiency was determined by RT-PCR. **b** HT-29 cells were treated with DMSO or TSZ for 4 h, and then incubated together with or without MG132 for another 4 h. The mRNA levels of the indicated genes were measured by RT-PCR (left). The cell viability was determined by CellTiter-Glo (right). MG132, 10 μM. **c** HT-29 cells expressing GFP or 3×HA-IκBα were treated as indicated. The cell lysates were collected for western blotting. **d** Western blotting analysis of lysates from 3×HA-IκBα-expressing HT-29 cells treated as indicated for 8 h. **e** MEFs stably expressing 3×HA-IκBα were treated as indicated for 4 h. The cell lysates were collected for western blotting analysis. T, 100 ng/ml TNFα; S, 100 nM SM-164; Z, 20 μM zVAD. **f** HT-29 cells were treated as indicated for 8 h. The cell lysates were immunoprecipitated with an anti-IκBα antibody. The immunoprecipitates and cell lysates were analyzed by western blotting with indicated antibodies. **g**, **h** WT MEFs (**g**) and MEFs stably expressing the indicated shRNAs (**h**) were treated as indicated for 4 h. The cell lysates were immunoprecipitated with an anti-IκBα antibody. The immunoprecipitates and cell lysates were analyzed by western blotting with indicated antibodies

The β-TrCP-mediated ubiquitination and the subsequent proteasomal degradation of IκBα is known to be the prerequisite for the p65/p50 translocation to nucleus^14–16,30^. Consistently, either knockdown of β-TrCP2 or the delayed addition of MG132 blocked *Cxcl8* and *Cxcl1* expression during necroptosis (Fig. 6a, b). However, no obvious difference of IκBα protein levels was found between cells treated with TSZ and TSZ plus necroptosis inhibitors (Fig. 5d and Supplementary Figure 7b), which might be due to newly synthesized IκBα by NF-κB. Indeed, the transcription of IκBα was induced during necroptosis in a RIPK1–RIPK3–MLKL-dependent manner (Supplementary Figure 8a–g).

We next expressed an exogenous IκBα lacking the NF-κB response element to directly monitor the protein turnover of IκBα. Similar to endogenous IκBα, the phosphorylation and degradation of exogenous IκBα occurred with two peaks; however, the protein level of exogenous IκBα was not restored in the second peak (Fig. 6c and Supplementary Figure 9a). In contrast, both waves of IκBα degradation were abolished by the S32A/S36A mutation in IKK-mediated phosphorylation sites as well as the K21R/K22R mutation in SCF^β-TrCP^-dependent ubiquitination sites (Supplementary Figure 9b). Furthermore, compared to that of TNFα or TS, necroptosis induced a more pronounced decrease of exogenous IκBα protein at the second peak, which could be blocked by necroptosis inhibitors as well as by the IKK inhibitor TPCA-1 (Fig. 6d, e). On the other hand, the first wave of IκBα degradation was blocked by TPCA-1 but not by necroptosis inhibitors (Supplementary Figure 9c–e), and TNFα alone induced IκBα degradation was not affected by NSA (Supplementary Figure 9f), indicating that the RIPK1 kinase activity, RIPK3, and MLKL were specifically required for the second wave of IκBα degradation.

Since IκBα was degraded through the proteasomal pathway after its ubiquitination, we characterized the ubiquitination of IκBα during necroptosis. We found that IκBα ubiquitination in cells treated with TSZ was significantly stronger than that induced by TNFα alone, which was blocked by IKK inhibition, and by Nec-1s, GSK872, NSA, or silencing MLKL (Fig. 6f–h). Overall, these results indicated that the RIPK1–RIPK3–MLKL axis promoted IκBα degradation mediated by ubiquitination.

### Necroptosis induced by MLKL oligomerization is weak in promoting cytokine expression

**Fig. 7:**
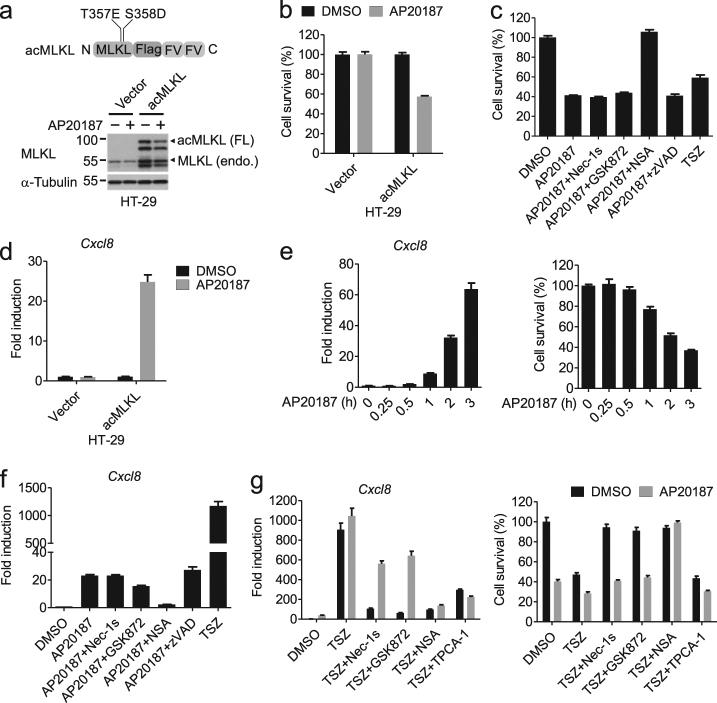
Necroptosis induced by forced dimerization of MLKL is much less effective in inducing cytokine production than that by TSZ. **a** Schematic representation of the oligomerizable MLKL (acMLKL) (upper) and western blotting analysis of lysates from HT-29 cells stimulated with DMSO or AP20187 for 2 h (lower). FL, full length; endo., endogenous. **b** HT-29 cells stably expressing the indicated constructs were stimulated with DMSO or AP20187 for 2 h. The cell viability was determined by CellTiter-Glo. **c** HT-29 cells expressing acMLKL were treated with the indicated compounds for 6 h except AP20187, which was administered in the last 2 h. The cell viability was determined by CellTiter-Glo. **d** HT-29 cells stably expressing the vector or acMLKL were stimulated with DMSO or AP20187 for 2 h. *Cxcl8* mRNA levels were measured by qPCR. **e** HT-29 cells stably expressing acMLKL were stimulated with AP20187 for the indicated time. The expression of *Cxcl8* was measured by qPCR (left). Cell viability was determined by CellTiter-Glo (right). **f** The acMLKL expressing HT-29 cells were treated with the indicated compounds for 6 h except AP20187, which was added during the last 2 h of incubation. The expression of *Cxcl8* was measured by qPCR. **g** HT-29 cells stably expressing acMLKL were treated as indicated for 8 h except AP20187. AP20187 was administered in the last 2 h. The expression of *Cxcl8* was measured by qPCR. The cell viability was determined by CellTiter-Glo. Data were represented as mean ± SEM of triplicates

Oligomerization of MLKL is essential for necroptosis. To assess whether MLKL oligomers are sufficient for cytokine induction during necroptosis, we constructed an induced-active (ac) form of MLKL (acMLKL) by fusing tandem dimerization domains, FKBP (F36V), to the C-terminus of the constitutively active human T357E/S358D double mutant, which could oligomerize upon the addition of the dimerizer, AP20187 (Fig. 7a). Indeed, the induction of MLKL dimerization led to considerable amount of cell death inhibitable by NSA, an inhibitor of MLKL oligomerization, but not by RIPK1 inhibitor Nec-1s, the RIPK3 inhibitor GSK872, or caspase inhibitor zVAD (Fig. 7b, c). We found that the induction of MLKL dimerization led to an increase in the mRNA levels of *Cxcl8* that was blocked by NSA but not Nec-1s, GSK872, or zVAD (Fig. 7d–f). The expression of *Cxcl8* induced by MLKL dimerization was much lower than that induced by TSZ (Fig. 7f), indicating that necroptosis execution induced by oligomerization of MLKL alone cannot account for the robust induction of cytokines by TSZ. However, TSZ-induced cytokine production in the presence of forced MLKL oligomerization was completely blocked by the IKK inhibitor TPCA-1 and the MLKL inhibitor NSA, and partially inhibited by Nec-1s and GSK872 (∼50%) (Fig. 7g). On the other hand, TNFα alone cannot enhance the cytokine expression in cells with MLKL oligomers (Supplementary Figure 10). Therefore, in addition to MLKL oligomers, RIPK1- and RIPK3-activated signaling events as well as IKK activation mediated by TNFα signaling may contribute to the robust cytokine expression during TSZ-induced necroptosis.

## Discussion

Necrosis is known to be proinflammatory from the passive leakage of DAMPs due to disrupted cytoplasmic membrane. However, the discovery of necroptosis, a regulated necrotic cell death mechanism, raised the question if there is an active program that can promote inflammation during necroptosis, i.e., inflammation beyond that activated by the passive release of intracellular content alone. Through a comparative transcriptome analysis of TNFα-induced necroptosis in the presence or absence of the RIPK1 kinase inhibitor Nec-1s, we discovered that necroptosis exerts a profound effect on transcription, activating the expression of a large number of genes. Notably, many of the upregulated genes, such as inflammatory cytokines, are in common with the target genes of TNFα pathway and all of them were expressed at a substantially higher level during necroptosis than that triggered by TNFα. These results suggest that necroptosis enhances TNFα-induced gene transcription. The greatly elevated cytokine expression appears to be a common feature of necroptosis as it was found in a panel of necroptotic cell lines and can be activated by different types of necroptotic stimuli including TNFα and TRAIL. The overrepresentation of TNFα target genes in the necroptosis-upregulated genes provides compelling evidence to show that necroptosis exacerbates TNFα-mediated inflammation. Although DAMPs can promote inflammation, we demonstrated that the increased cytokine production in TNFα-induced necropotosis is independent of DAMPs release and requires cell-intrinsic activation of NF-κB and p38. Future studies will be needed to explore the transcriptional regulatory mechanisms that control the induction of proinflammatory cytokine expression by necroptosis.

We showed that cytokine production mediated by MLKL oligomerization alone was substantially lower than that induced by TSZ. This result suggests that MLKL oligomers are necessary but insufficient for maximum cytokine production in necroptosis and events upstream of MLKL oligomerization are required. Mechanistically, we propose that the RIPK1 kinase–RIPK3–MLKL axis does not induce the activity of the IKK complex, but rather activates p38, and promotes the ubiquitination and degradation of IκBα that could activate NF-κB. Consistently, a much higher level of ubiquitinated IκBα is induced by TSZ than that by TNFα. Therefore, the necroptosis machinery likely acts downstream of IKK to sustain the activation of NF-κB. However, as we were not able to detect an association of RIPK1, RIPK3, or MLKL with IκBα, necroptosis might promote IκBα degradation via an indirect mechanism. Taken together, we propose that TNFα-mediated necroptosis dependent on RIPK1, RIPK3, and MLKL activation triggers enhanced inflammatory cytokine gene transcription through a cell-autonomous mechanism involving NF-κB and p38. However, we do not exclude that other mechanisms might also exist in necroptotic cells underlying the cell-intrinsic activation of cytokine expression.

Studies in mouse models of human diseases have demonstrated an important role of necroptosis in a variety of diseases including inflammatory diseases, neurodegeneration, and cancer^31–35^. As a result, RIPK1 kinase inhibitors have been advanced into the human clinical trial for the treatment of colitis, amyotrophic lateral sclerosis, and Alzheimer’s diseases^36,37^. Our study suggests that the enhancement of NF-κB activation by necroptosis could contribute to the pathogenesis by mediating the production of proinflammatory cytokines, which may serve as biomarkers to monitor the efficacy of therapeutic interventions by inhibition of RIPK1.

## Materials and methods

### Reagents and antibodies

Recombinant human TNFα (Cat# C008) was purchased from Novoprotein Scientific Inc. (Summit, NJ, USA). Recombinant human TRAIL (Cat# 375-TL) was obtained from R&D Systems (Minneapolis, MN, USA). MG132 and zVAD were purchased from Selleckchem (Houston, TX, USA). GSK872 and NSA were purchased from Calbiochem (San Diego, CA, USA). 5Z-7-Oxozeaenol was purchased from Sigma-Aldrich (St. Louis, MO, USA). TPCA-1, SP600125, PH-797804, and AP20187 were purchased from MedChemexpress (Princeton, NJ, USA). R-7-Cl-O-Nec-1 (Nec-1s) and SM-164 were custom synthesized.

Antibodies used in the study were IκBα (C-21, Cat# sc-371), p65 (C-20, Cat# sc-372), Ub (P4D1, Cat# sc-8017), NEMO (FL-491, Cat# sc-8330), and GFP (FL, Cat# sc-8334) from Santa Cruz (Dallas, TX, USA); IκBα (Cat# 4814), p-IκBα (Ser32, Cat# 2859), p65 (Cat# 8242), p-p65 (Ser536, Cat# 3033), IKKβ (Cat# 8943), RIPK1 (Cat# 3493), and p38 (Cat# 9212) from Cell Signaling (Danvers, MA, USA); FLAG (Cat# F3040) from Sigma-Aldrich (St Louis, MO, USA); CXCL8 (Cat# 17038-1-AP) from Proteintech (Chicago, IL, USA); and α-Tubulin (Cat# PM054) from MBL (Woburn, MA, USA). Antibodies including RIPK3 (Cat# ab72106), MLKL (Cat# ab183770), and p-MLKL (Ser358, Cat# ab187091) were obtained from Abcam (Cambridge, UK) to detect human proteins. Anti-RIPK3 and anti-MLKL antibodies were produced in-house to probe the mouse proteins. HRP conjugated goat anti-rabbit IgG (H+L) secondary antibody (Cat# 31460) and HRP conjugated goat anti-mouse IgG (H+L) secondary antibody (Cat# 31430) were purchased from Thermo Fisher Scientific (Waltham, MA, USA).

### Cell culture and treatments

HT-29 cells were cultured in McCoy’s 5A medium (GIBCO, Grand Island, NY, USA), supplemented with 10% FBS (Biowest, Nuaillé, France) and 1% penicillin and streptomycin. MEFs, HEK293T, HT-22, and L929 cells were cultured in DMEM (GIBCO) supplemented as described above. Jurkat cells were cultured in RPMI 1640 medium (GIBCO) supplemented as described above. Cells were cultured at 37 °C in a humidified atmosphere with 5% CO_2_.

For HT-29, L929, and HT-22 cells, TNFα and SM-164 were used at a concentration of 10 ng/ml and 50 nM, respectively. For MEFs, TNFα and SM-164 were used at a concentration of 50 ng/ml and 100 nM, respectively; 100 ng/ml TNFα was used to treat wild-type and FADD-deficient Jurkat cells. SM-164 was used at a concentration of 100 nM to treat wild-type Jurkat cells. Nec-1s, GSK872, NSA, zVAD, 5Z-7, TPCA-1, and AP20187 were used at 10 μM, 10 μM, 2 μM, 20 μM, 2.5 μM, 10 μM, and 20 nM, respectively. TRAIL were used at a concentration of 100 ng/ml. Identical concentrations were used as describe above, unless otherwise stated. DMSO (<0.2%) was used as a vehicle-only control.

### Plasmid construction

Human and mouse IκBα were generated by PCR amplification from the cDNA of HT-29 cells and MEFs, respectively, and then cloned into the pMSCV vector or a lentiviral vector made in-house. The S32A/S36A double mutant or K21R/K22R double mutant IκBα were created by PCR-based site-directed mutagenesis. To construct the dimerizable MLKL, human MLKL and FKBP were amplified from cDNA of HT-29 cells. T357E/S358D MLKL and F36V FKBP were generated through PCR-based site-directed mutagenesis. The mutated MLKL, F36V FKBP, and Flag tag oligonucleotides were cloned into pMSCV vector using ClonExpress^TM^ One Step Cloning Kit (Vazyme, Nanjing, China). The annealed shRNA and single-guide RNA (sgRNA) oligonucleotides were cloned into the pLKO.1 vector and LentiCRISPR v2 vector, respectively. All plasmids were confirmed by sequencing.

The shRNAs used in this study were selected from the RNAi Consortium (TRC) (Broad Institute, MA, USA) and are listed in Supplementary Table 1. To silence RIPK1 in MEFs, the following sgRNAs were used: sgRIPK1 #1_Sense (S): caccgGGGTCTTTAGCACGTGCATC, sgRIPK1 #1_Antisense (AS): aaacGATGCACGTGCTAAAGACCCc; sgRIPK1 #2_S: caccgAGAAGAAGGGAACTATTCGC, sgRIPK1 #2_AS: aaacGCGAATAGTTCCCTTCTTCTc; sgRIPK1 #3_S: caccgTGTGAAAGTCACGATCAACG, sgRIPK1 #3_AS: aaacCGTTGATCGTGACTTTCACAc.

### Virus packaging and transduction

Recombinant lentivirus and retrovirus were packaged in HEK293T cells in the presence of packaging plasmids. Specifically, one well of HEK 293T cells in 6-well plates was transfected with 2 μg plasmids including the expression vector and the packaging plasmids. Seventy-two hours later, the media containing the virus were collected and filtered through a 0.45-μM PVDF membrane (Millipore, Billerica, MA, USA). Transduction was performed by incubating cells with the virus-containing media in the presence of 8 μg/ml Polybrene for 48 h. To establish the acMLKL-expressing HT-29 cells or stable cell lines expressing shRNA or sgRNA, cells were selected with 5 μg/ml puromycin for 3–4 days after virus transduction.

### RNA interference

HT-22 cells were transfected with 50 nM siRNA using Lipofectamin RNAiMax (Invitrogen) following the manufacturer’s instruction. The sense sequence of mouse MLKL siRNA is GAGAUCCAGUUCAACGAUA.

### Quantitative PCR  (qPCR) and semi-quantitative reverse  transcription PCR (RT-PCR) analysis

Total RNA was extracted and purified using Trizol Reagent (Thermo Fisher Scientific). Reverse transcription reactions were performed with the M-MLV reverse transcriptase (TaKaRa, Otsu, Japan). For qPCR, SYBR Green Master Mix (Vazyme) was used in StepOnePlus PCR Systems (Applied Biosystems) and the PCR conditions were 95 °C for 10 min, and 40 cycles of 95 °C for 15 s and 60 °C for 30 s. Fold induction of gene expression is calculated using the ΔΔCt method^38^. Namely, cycle threshold (Ct) values for genes of interest were normalized to *C*_T_ values of *Gapdh* (human) or *β-Actin* (mouse) and then to that of control groups. For semi-quantitative PCR, identical reactions with polymerase (Vazyme) were performed to compare the expression level of indicated genes. PCR primers used in this study are listed in Supplementary Table 2.

### polyA-selected RNA-seq

HT-29 cells were treated with DMSO, 10 ng/ml TNFα, or the combination of 10 ng/ml TNFα, 50 nM SM-164, and 20 μM zVAD in the presence or absence of 10 μM Nec-1s for 8 h. HT-29 cells then were homogenized in a 1.5-ml tube containing 1 ml of Trizol Reagent (Thermo Fisher Scientific). RNA isolation was followed in accordance with manufacturer’s instruction. RNA was resuspended in DEPC-treated RNase free water (Thermo Fisher Scientific). TURBO DNA free kit was used to remove residual DNA contamination according to manufacturer’s instruction (Thermo Fisher Scientific); 1 μg of total RNA was used for sequencing library preparation. PolyA-tailed RNAs were selected by mRNA Capture Beads (VAHTS), followed by the mRNA-seq V2 Library Prep Kit for Illumina according to manufacturer’s instruction (VAHTS). The library quality was examined by Bioanalyzer 2100 (Agilent). The libraries were pooled together in equimolar amounts to a final 2 nM concentration. The normalized libraries were denatured with 0.1 M NaOH (Sigma). Pooled denatured libraries were sequenced on the illumina NextSeq 550 with single end 150 bps.

### RNA-seq analysis

Sequencing reads were mapped to the reference genome hg38 with STAR2.3.0e by default parameter^39^. The read counts for each gene were calculated by HTSeq-0.5.4e htseq-count with parameters “-m intersection-strict -s no”^40,41^. The count files were used as an input to R package DESeq for normalization.

### Immunoprecipitation

After stimulation, the media were removed and plates were placed on ice and flooded with ice-cold PBS to stop stimulation. Cells were then lysed on ice in NP40 lysis buffer (20 mM Tris-HCl pH 7.5, 150 mM NaCl, 1 mM EDTA, 1% NP40, 10% Glycerol, 3 mM NaF, 1 mM β-glycerophophate) supplemented with the protease inhibitor cocktail (Biotool, Houston, TX, USA). Cell lysates were incubated on ice for 10 min, and clarified at 15,000×*g* for 15 min at 4 °C. Lysates were then incubated with corresponding antibodies overnight, followed by incubation with the Protein G Agarose (Thermo Fisher Scientific) for 4 h at 4 °C. After four washes with NP40 lysis buffer, the beads were boiled in SDS loading buffer (50 mM Tris-HCl pH 6.8, 2% SDS, 10% glycerol, and 100 mM DTT) for 5 min.

### Western blotting

Cell extracts or immunoprecipitates in SDS loading buffer were subjected to SDS-PAGE. After transfer of the resolved proteins to PVDF membrane (Millipore), the membranes were blocked with 5% reconstituted dry milk in 0.1% Tween Tris-buffered saline (TBS-T) for 30 min at room temperature, and then successively incubated with primary antibodies overnight at 4 °C and the peroxidase-coupled secondary antibodies for 1 h at room temperature. Proteins were visualized by enhanced chemiluminescence substrate.

### In vitro kinase assay

HT-29 cells were lysed in NP40 lysis buffer. Immunoprecipitation was performed with an anti-NEMO antibody as described above. Following three washes with 20 mM Tris-HCl pH 7.5, the immunoprecipitated IKK complex was incubated with 0.01 mg/ml GST-IκBα (20–217) (Cat# ag12979, Proteintech) in a kinase buffer (25 mM Tris-HCl pH 7.5, 10 mM MgCl_2_, 50 μM ATP, 2 mM DTT, 5 mM β-glycerophosphate, and 0.1 mM Na_3_VO_4_) at 30 °C for 1 h. The reaction mixtures were then subjected to SDS-PAGE. The resulted phospho-GST-IκBα was detected by an anti-p-IκBα antibody (Ser32, Cat# 2859, Cell Signaling).

### Viability assay

Cell survival was determined by the CellTiter-Glo Luminescent Cell Viability Assay (Promega, Madison, WI, USA) following the manufacturer’s instruction. All cell survival assays were performed in triplicates.

### Flow cytometry

The mixed cultures of mCherry and GFP-labeled HT-29 cells were stimulated with DMSO or 10 ng/ml TNFα (T), 50 nM SM-164 (S), and 20 μM zVAD (Z) for 6 h. The cells were then trypsinized and separated by an Influx Cytometer (BD Biosciences) based on the mCherry and GFP expression.

### Immunocytochemistry

HT-29 cells were plated on coverslips and treated as indicated. Cells were fixed with 4% paraformaldehyde and permeabilized with 0.2% Triton X-100 in PBS. Cells were then incubated with 5% bovine serum albumin in PBS for 30 min and the anti-p65 antibody (C-20, Cat# sc-372, Santa Cruz) overnight at 4 °C, followed by three PBS washes and subsequent incubation with the fluorescent secondary antibody for 1 h at room temperature. DAPI (300 nM) (Thermo Fisher Scientific) was included in the final wash for nuclei counterstain. Cells were mounted with 10% glycerol and images were acquired with a laser scanning microscope (Leica SP8 Confocal System).

### Sample preparation for mass spectrometry

HT-29 cells were treated with DMSO or 10 ng/ml TNFα (T), 50 nM SM-164 (S), and 20 μM zVAD (Z) for 4 h. After three washes with PBS, the culture medium was replaced with DMEM and cells were incubated for another 8 h. The media were collected and cleared of cell debris by centrifugation. Secreted proteins in the supernatant of HT-29 culture media were concentrated with a 3-kDa centrifugal filter.

### Mass spectrometry

Two-hundred micrograms of proteins from the supernatant of cell culture were trypsin digested. The resulted peptides were analyzed on Thermo Scientific Q Exactive hybrid quadrupole-Orbitrap mass spectrometer. The protein identification and quantification were done by MaxQuant^42^. The tandem mass spectra were searched against UniProt human protein database and a set of commonly observed contaminants. The precursor mass tolerance was set as 20 ppm, and the fragment mass tolerance was set as 0.1 Da. The cysteine carbamidomethylation was set as a static modification, and the methionine oxidation was set as a variable modification. The FDR at peptide spectrum match level and protein level were controlled below 1%. The unique peptides plus razor peptides were included for label-free quantification in MaxQuant^43^.

### GEO accession

RNA-seq data can be downloaded from the GEO under accession number GSE 108621.

### Statistics

All statistical analyses were performed using the GraphPad Prism 5. All data points are shown as mean ± SEM unless otherwise stated. All experiments were repeated 2–3 times.

## Electronic supplementary material

supplementary Figure legends

Supplementary figures

Supplementary tables
