# Supplementary material for: Necroptosis promotes cell-autonomous activation of proinflammatory cytokine gene expression
Source: Cell Death Dis. 2018 Apr 27;9(5):500. doi: 10.1038/s41419-018-0524-y (PMC5923285; doi:10.1038/s41419-018-0524-y)
Supplement: Supplementary file 1 — supplementary Figure legends [file 41419_2018_524_MOESM1_ESM.docx]

**Supplementary Figure legends**

**Supplementary Figure 1. Induction of cytokines by necroptosis in different cell lines.** (a) Detection of *Tnfα*, *Cxcl8* and *Gapdh* gene expression in HT-29 cells after the indicated treatment by RT-PCR. (b) MEFs were treated with DMSO or TSZ for 4 h. The levels of the indicated transcripts were determined by qPCR and were expressed as fold induction by TSZ over that of untreated groups. (c) Jurkat cells were treated with TSZ for the indicated time. The expression of *Cxcl8* and *Gapdh* was measured by RT-PCR (left). The cell viability was determined by CellTiter-Glo (right). (d-e) HT-22 cells (d) and L929 cells (e) were treated with TSZ for the indicated time. The expression of *Cxcl2*, *Csf2* and *β-Actin* was measured by RT-PCR (left). The cell viability was determined by CellTiter-Glo (right). (f) Detection of *Cxcl2*, *Csf2* and *β-Actin* gene expression in MEFs after the indicated treatment by RT-PCR. (g) FADD deficient Jurkat cells (Jurkat FADD def.) were treated as indicated. The expression of *Cxcl8*, *Tnfα* and *Gapdh* was measured by RT-PCR (left). The cell viability was determined by CellTiter-Glo (right). (h-i) HT-29 cells (h) or Jurkat cells (i) were treated as indicated for 12 h (h), or 18h (i). The expressions of the cytokine were determined by RT-PCR (left). The cell viability was determined by CellTiter-Glo (right). Data were presented as mean ± SEM of triplicates.

**Supplementary Figure 2. The transcriptional induction of cytokines by necroptosis.** HT-29 cells were treated as indicated for 8 h. The mRNA levels of *Cxcl8*, *Cxcl1*, *Cxcl2*, *Tnfα* and *Gapdh* were measured by RT-PCR. The cell viability was determined by CellTiter-Glo. Data were represented as mean ± SEM of triplicates. Act D, 10 μM actinomycin D.

**Supplementary Figure 3. NF-κB machinery is essential for the induction of cytokines during necroptosis.** (a) MEFs were treated with TSZ for the indicated time. The cell lysates were collected for western blotting analysis. (b) Jurkat cells were treated as indicated for 24 h. The mRNA levels of *Cxcl8* and *Gapdh* were measured by RT-PCR (left). The cell viability was determined by CellTiter-Glo (right). (c) HT-22 cells were treated as indicated. The mRNA levels of *Cxcl2*, *Csf2* and *β-Actin* were measured by RT-PCR after 4 h of treatment (left). The cell viability was determined by CellTiter-Glo after 6 h of treatment (right). (d) L929 cells were treated as indicated. The mRNA levels of *Cxcl2*, *Csf2* and *β-Actin* were measured by RT-PCR after 2.5 h of treatment (left). The cell viability was determined by CellTiter-Glo after 3 h of treatment (right). (e) HT-29 cells were pre-treated with DMSO or TSZ for 2 h, followed by incubation with or without 5Z-7 for another 6 h. The amounts of *Cxcl8* and *Cxcl1* transcripts were determined by qPCR. (f-g) MEFs were pre-treated with DMSO or TSZ for 1 h, followed by incubation with DMSO, 5Z-7 or TPCA-1 for another 3 h. The expression of *Cxcl2* and *Csf2* was measured by qPCR (f). The cell lysates were collected for western blotting analysis (g). (h-i) TPCA-1 was added at the indicated time points after the addition of TSZ (t=0) to HT-29 cells. At 8 h, the amounts of transcripts of *Cxcl8* and *Cxcl1* were determined by qPCR (h) and the cell lysates were subjected to western blotting (i). Data were represented as mean ± SEM of triplicates.

**Supplementary Figure 4. MAPKs and Noncanonical NF-κB are not required for cytokine induction by TSZ.** (a) HT-29 cells were treated as indicated for 8 h. The mRNA levels of *Cxcl8* and *Cxcl1* were measured by qPCR. The cell viability was determined by CellTiter-Glo. SP600125, 10 μM, JNK inhibitor; PH-797804, 10 μM, p38 inhibitor. (b) HT-29 cells stably expressing shRNA targeting p38α or control shRNA were treated as indicated. The mRNA levels of *Cxcl8*, *Cxcl1* and *Cxcl2* were measured by qPCR after 8 h of treatment and the cell viability was determined by CellTiter-Glo after 24 h of treatment(right). The knockdown efficiency was determined by western blotting. (c) HT-29 cells stably expressing RELB shRNA or control shRNA were treated as indicated. The mRNA levels of *Cxcl8* and *Cxcl1* were measured by qPCR after 8 h of treatment. The cell viability was determined by using Celltiter-Glo after 36 h of treatment. The knockdown efficiency was determined by RT-PCR. Data were represented as mean ± SEM of triplicates.

**Supplementary Figure 5.** **Necroptotic machinery is required for the induction of cytokine**. (a) Jurkat cells were treated as indicated for 24 h. The mRNA levels of *Cxcl8* and *Gapdh* were measured by RT-PCR (left). The cell viability was determined by CellTiter-Glo (right). (b) HT-22 cells were treated as indicated. The mRNA levels of *Cxcl2*, *Csf2* and *β-Actin* were measured by RT-PCR after 4 h of treatment (left). The cell viability was determined by CellTiter-Glo after 6 h of treatment (right). (c) L929 cells were treated as indicated. The mRNA levels of *Cxcl2*, *Csf2* and *β-Actin* were measured by RT-PCR after 2.5 h of treatment (left). The cell viability was determined by CellTiter-Glo after 3 h of treatment (right). (d) FADD deficient Jurkat cells were treated with DMSO, TNFα (T), Nec-1s, GSK872 or NSA as indicated for 6 h. The mRNA levels of *Cxcl8*, *Tnfα* and *Gapdh* were measured by RT-PCR (left). The cell viability was determined by CellTiter-Glo (right). (e) MEFs expressing control sgRNA or sgRNA targeting RIPK1 were treated as indicated. The mRNA levels of *Cxcl2* and *Csf2* were measured by qPCR after 4 h of treatment. The cell viability was determined by CellTiter-Glo after 26 h of treatment. The knockdown efficiency was determined by western blotting. (f-g) HT-22 cells were transfected with control siRNA or MLKL siRNA for 72 h. Then the cells were treated with DMSO or TSZ for 6 h. The expression of *Cxcl2*, *Csf2* and *β-Actin* was determined by RT-PCR (left) and the cell viability was determined by CellTiter-Glo (right) (f). The cell lysates were collected for western botting analysis (g). (h) L929 cells transfected with control shRNA or shRNA targeting MLKL were treated with DMSO or TSZ for 3 h. The mRNA levels of *Cxcl2*, *Csf2* and *β-Actin* were measured by RT-PCR. The cell viability was determined by CellTiter-Glo. The knockdown efficiency was determined by western blotting. Data were represented as mean ± SEM of triplicates.

**Supplementary Figure 6.** **Necroptosis machinery is dispensable for the cytokine production induced at the early time of TSZ treatment, as well as by TNFα alone or TS.** (a) Quantitative PCR analysis of *Cxcl8* mRNA levels in HT-29 cells treated as indicated. (b) Detection of *Cxcl2*, *Csf2* and *β-Actin* gene expression in MEFs after the indicated treatment by RT-PCR. (c-d) Quantitative PCR analysis of *Cxcl8* and *Cxcl1* mRNA levels in HT-29 cells after the indicated treatment for 8 h. (e) HT-29 cells stably expressing the control shRNA or MLKL shRNA were treated as indicated. The mRNA levels of *Cxcl8* and *Cxcl1* were measured by qPCR after 8 h of treatment. The cell viability was determined by CellTiter-Glo after 24 h of treatment. Data were represented as mean ± SEM of triplicates.

**Supplementary Figure 7. RIPK1 kinase activity, RIPK3 and MLKL are dispensable for phosphorylation of p65 and IκBα induced by TSZ.** (a) Western blotting of lysates from MEFs treated as indicated. (b) MEFs were treated as indicated for 4 h. The cell lysates were collected for western blotting analysis. (c) Western blotting of lysates from HT-29 cells treated as indicated. (d-e) HT-29 cells stably expressing the indicated shRNA were treated with DMSO or TSZ for 8 h. The cell lysates were collected for western blotting. (f) MEFs stably expressing the indicated shRNA were stimulated with TSZ for 4 h. The expression of the indicated protein was measured by western blotting.

**Supplementary Figure 8. The expression of *Iκbα* is induced during necroptosis.** (a) HT-29 cells were treated with TSZ for the indicated time. The expression of *Iκbα* was measured by qPCR. (b) MEFs were treated for the indicated periods of time with TSZ. The expression of *Iκbα* was determined by qPCR. (c) HT-29 cells were treated as indicated for 8 h. The expression of *Iκbα* was determined by qPCR. (d) MEFs were treated as indicated for 4h. The expression of *Iκbα* was measured by qPCR. (e-g) HT-29 cells expressing the indicated shRNA (e, g) or WT HT-29 cells (f) were treated as indicated for 8 h. The expression of *Iκbα* was measured by qPCR. Data were represented as mean ± SEM of triplicates.

**Supplementary Figure 9. Degradation of IκBα is promoted by necroptosis.** (a) MEFs transfected with GFP or 3xHA-IκBα were treated with TSZ for the indicated periods of time. The cell lysates were collected for western blotting. (b) HT-29 cells harboring the mutant forms of IκBα, the S32A/S36A double mutant or the K21R/K22R double mutant, were stimulated with TSZ. The cell lysates were collected at the indicated time for western blotting. (c) HT-29 cells stably expressing 3xHA-IκBα were treated as indicated for 15 min. The cell lysates were collected for western blotting. (d) MEFs stably expressing 3xHA-IκBα were treated as indicated for 15 min. The cell lysates were collected for western blotting. T: 100 ng/ml TNFα; S: 100 nM SM-164; Z: 20 μM zVAD. (e-f) HT-29 cells stably expressing 3xHA-IκBα were treated as indicated. The cell lysates were collected for western blotting analysis (left). The abundance of 3xHA-IκBα was quantified by densitometry, normalized to α-Tubulin using ImageJ and expressed as mean ± SEM of triplicates (right). s.e., short exposure; l.e., long exposure.

**Supplementary Figure 10. TNFα alone is unable to enhance cytokine production induced by MLKL dimerization.** HT-29 cells stably expressing the indicated constructs were pre-treated with or without TNFα for 6 h, and then treated with or without AP20187 for another 2h. The expression of cytokines was measured by RT-PCR (left). The cell viability was determined by CellTiter-Glo (right).
