# Supplementary material for: Necroptosis promotes cell-autonomous activation of proinflammatory cytokine gene expression
Source: Cell Death Dis. 2018 Apr 27;9(5):500. doi: 10.1038/s41419-018-0524-y (PMC5923285; doi:10.1038/s41419-018-0524-y)

Supplementary Figure 1

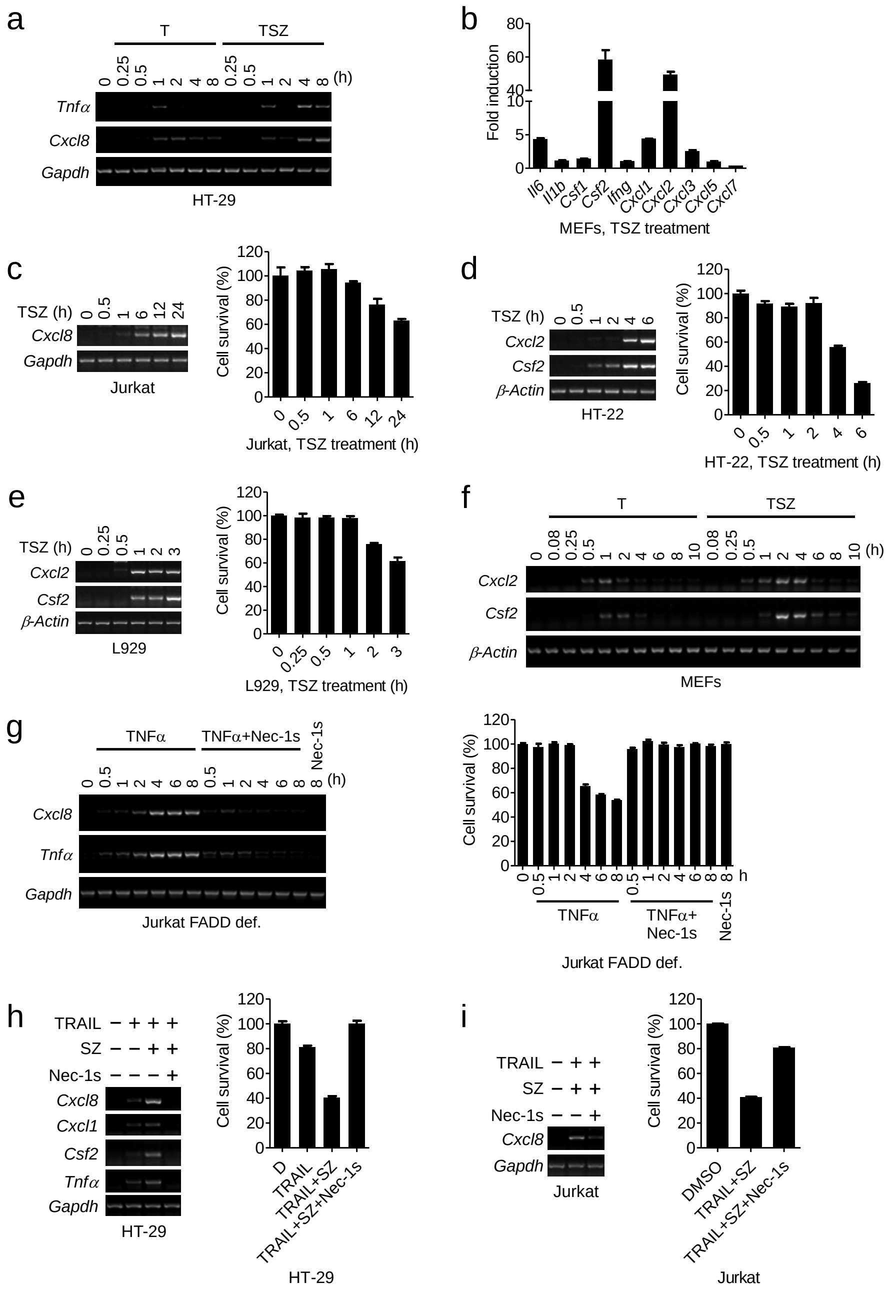

Supplementary Figure 2

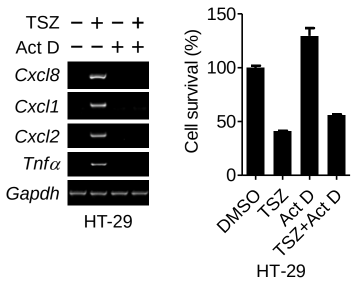

Supplementary Figure 3

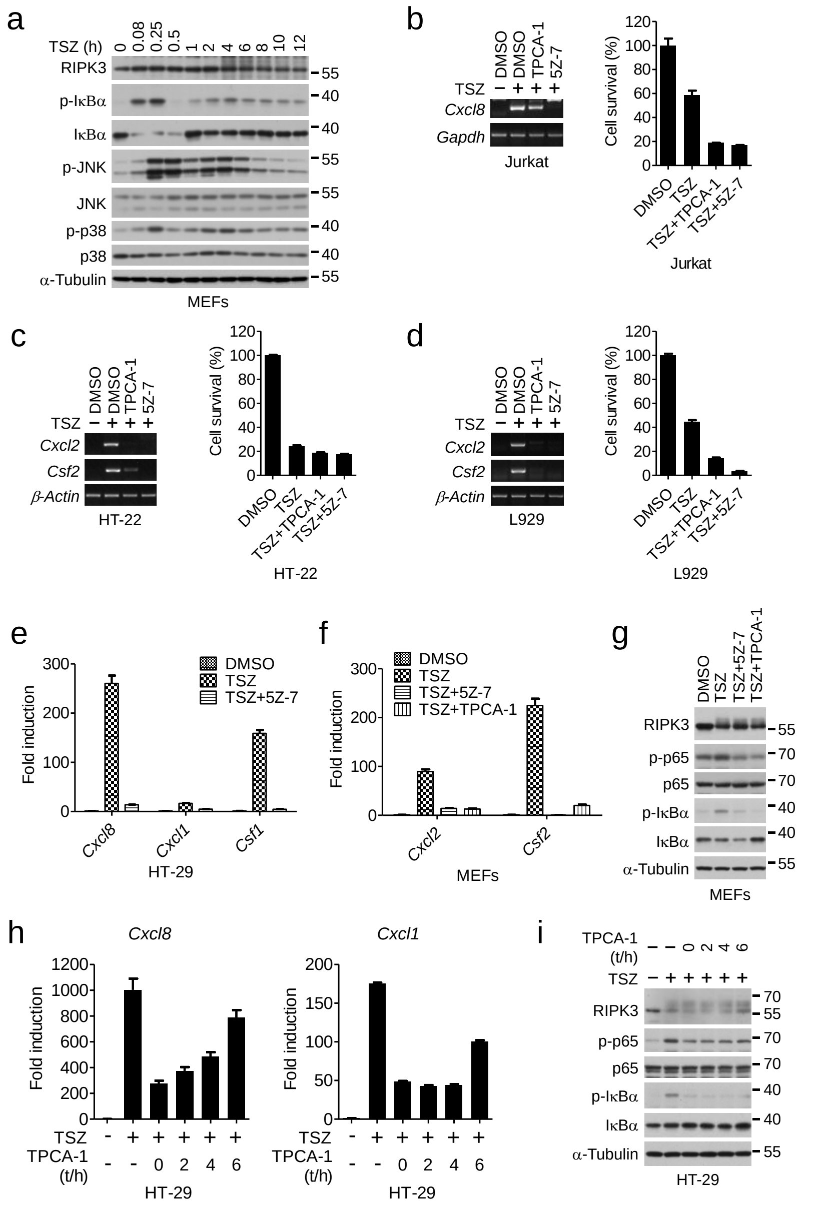

Supplementary Figure 4

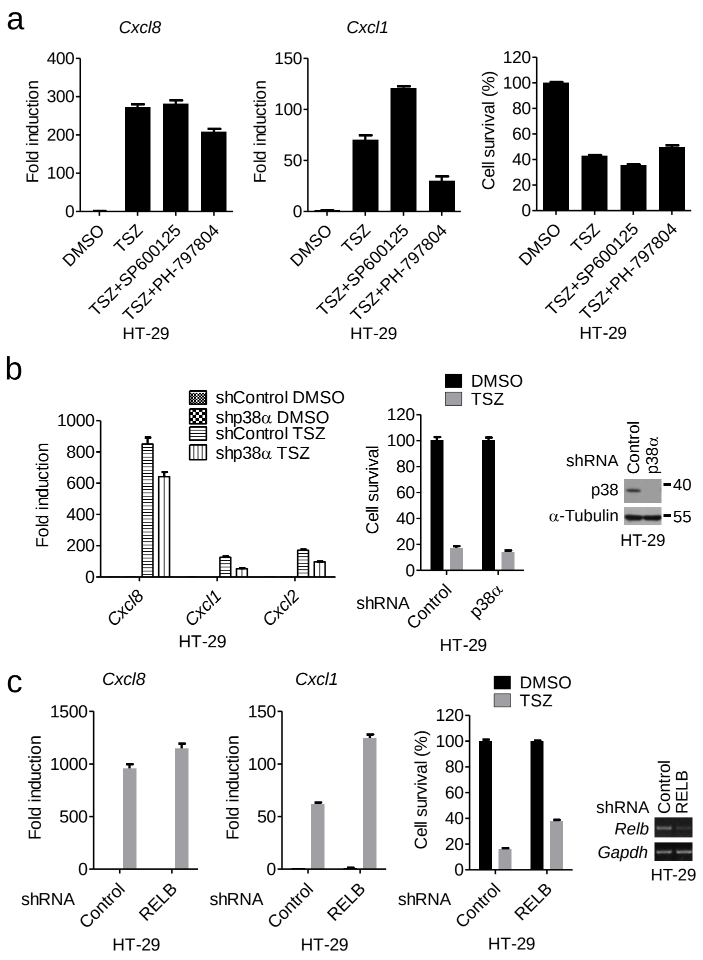

Supplementary Figure 5

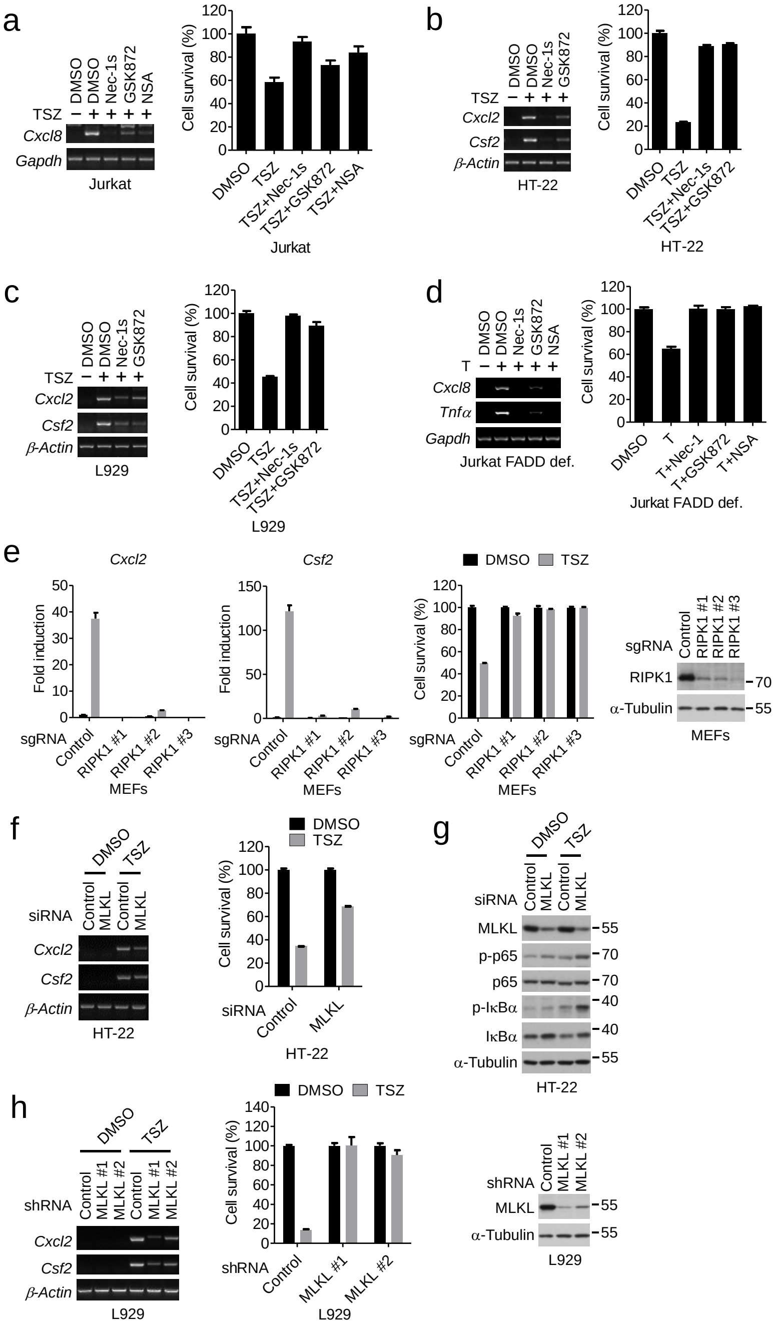

Supplementary Figure 6

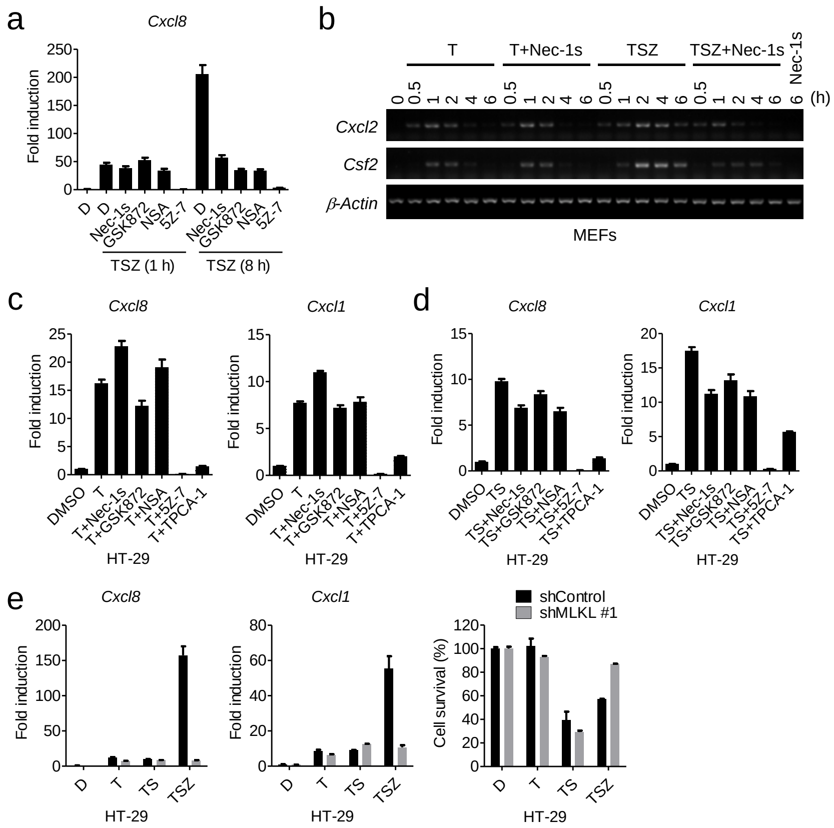

Supplementary Figure 7

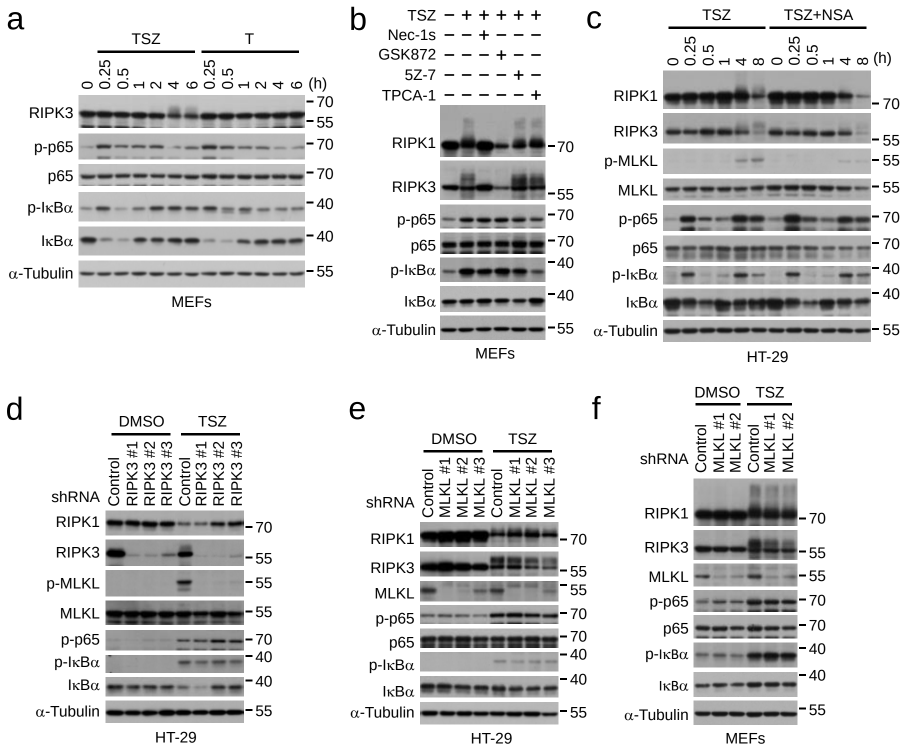

Supplementary Figure 8

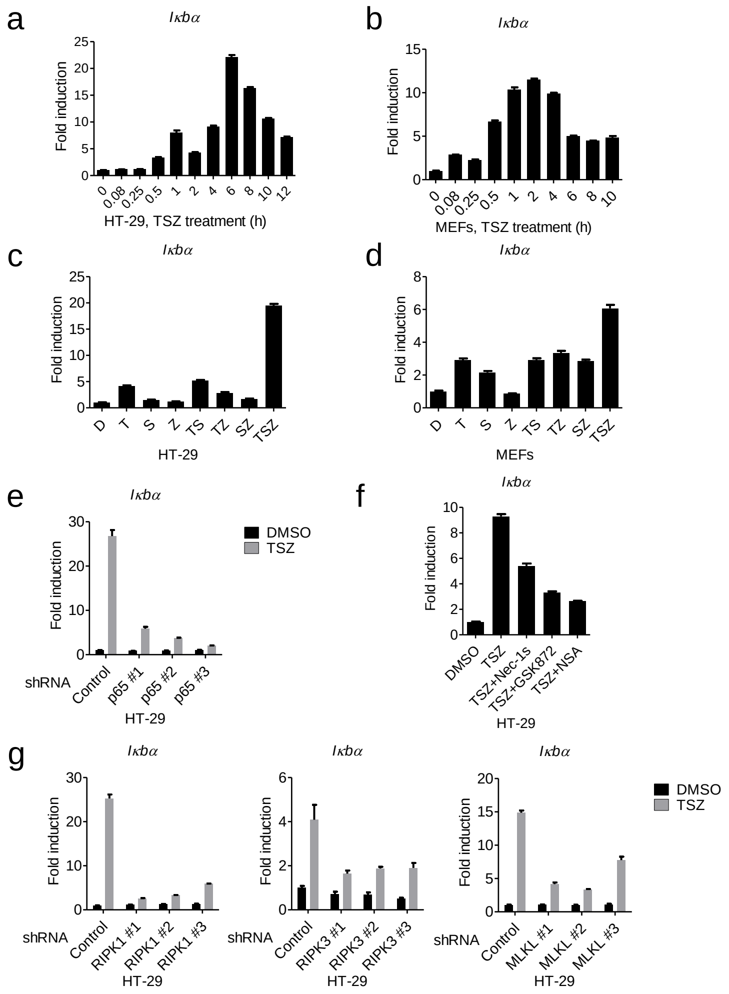

Supplementary Figure 9

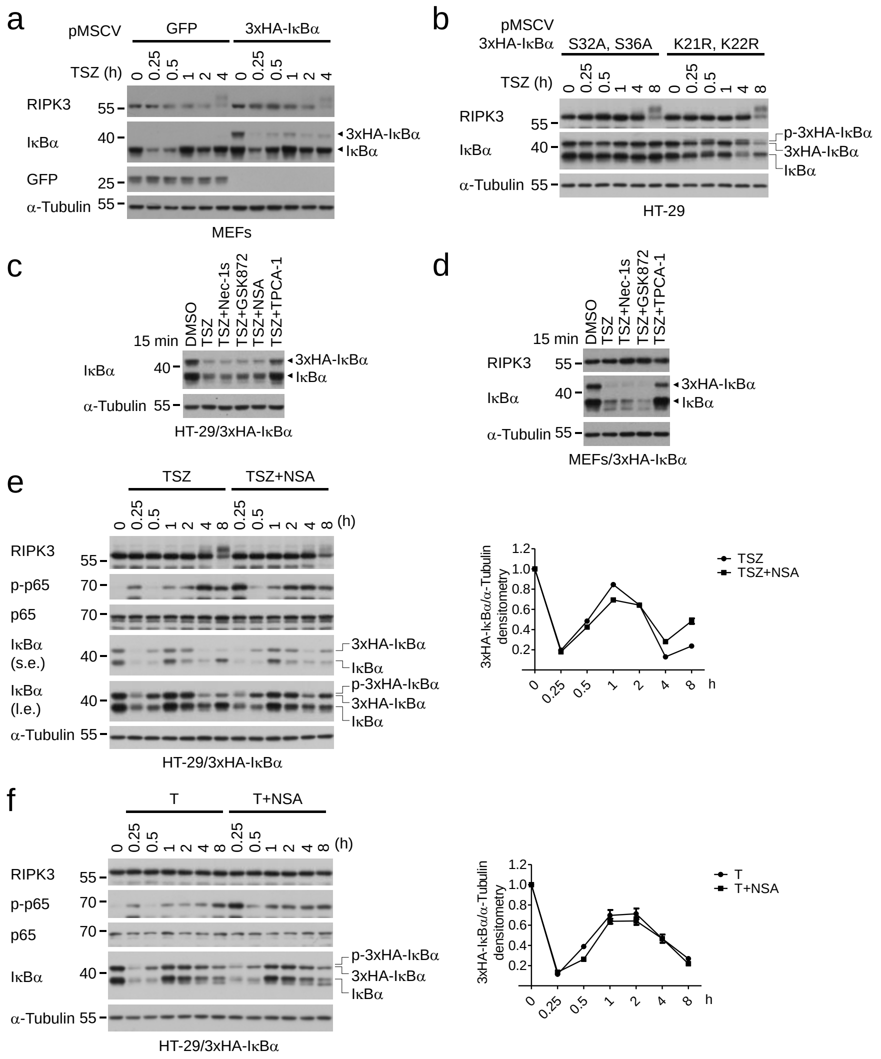

Supplementary Figure 10

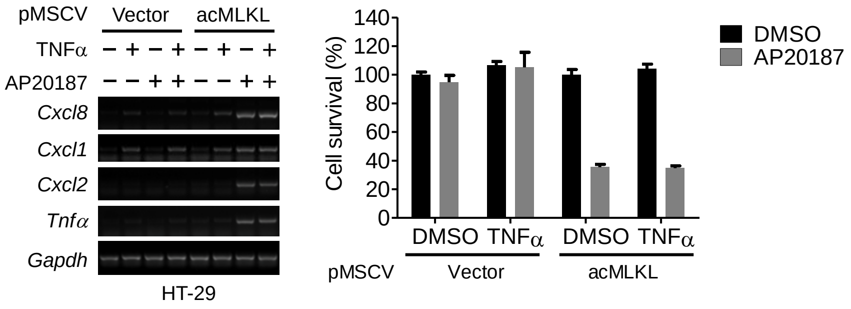

Supplement: Supplementary file 2 — Supplementary figures [file 41419_2018_524_MOESM2_ESM.pdf]
