# Supplementary material for: Necroptosis promotes cell-autonomous activation of proinflammatory cytokine gene expression
Source: Cell Death Dis. 2018 Apr 27;9(5):500. doi: 10.1038/s41419-018-0524-y (PMC5923285; doi:10.1038/s41419-018-0524-y)
Supplement: Supplementary file 3 — Supplementary tables [file 41419_2018_524_MOESM3_ESM.pdf]

Supplementary Table 1. Short hairpin RNA (shRNA) used in this study.

shRNA	Species	Sense sequence (5'-3')
RIPK1 #1	human	CCGGGTAGTACTCTGGGCGATATTTCTCGAGAAATATCGCCCAGAGTACTACTTTTTG
RIPK1 #2	human	CCGGGCAAAGACCTTACGAGAATTTCTCGAGAAATTCTCGTAAGGTCTTTGCTTTTTG
RIPK1 #3	human	CCGGGACGCCTCACTTAGTGGATAACTCGAGTTATCCACTAAGTGAGGCGTCTTTTTG
RIPK3 #1	human	CCGGGGCGACCGCTCGTTAACATATCTCGAGATATGTTAACGAGCGGTCGCCTTTTTG
RIPK3 #2	human	CCGGCTACAGCTTCGGGATCCTAATCTCGAGATTAGGATCCCGAAGCTGTAGTTTTTG
RIPK3 #3	human	CCGGAGGCCACAGGGTTGGTATAATCTCGAGATTATACCAACCCTGTGGCCTTTTTG
MLKL #1	human	CCGGGAGTCAAATCTACAGCATATCCTCGAGGATATGCTGTAGATTTGACTCTTTTTG
MLKL #2	human	CCGGCCTTCGGCATTGGGTTATCTACTCGAGTAGATAACCCAATGCCGAAGGTTTTTG
MLKL #3	human	CCGGGTGACTTGATTTGATCAATAGCTCGAGCTATTGATCAAATCAAGTCACTTTTTG
p65 #1	human	CCGGCGGATTGAGGAGAAACGTAAACTCGAGTTTACGTTTCTCCTCAATCCGTTTTTG
p65 #2	human	CCGGAGAGGACATTGAGGTGTATTTCTCGAGAAATACACCTCAATGTCCTCTTTTTTG
p65 #3	human	CCGGATGGATTCATTACAGCTTAATCTCGAGATTAAGCTGTAATGAATCCATTTTTTG
p38α	human	CCGGGTACTTCCTGTGTACTCTTTACTCGAGTAAAGAGTACACAGGAAGTACTTTTTG
RELB	human	CCGGCATGCTTCTGAAGTGGACATACTCGAGTATGTCCACTTCAGAAGCATGTTTTTG
β-TrCP2 #1	human	CCGGACTCTTCAAGTACCGACATTTCTCGAGAAATGTCGGTACTTGAAGAGTTTTTTG
β-TrCP2 #2	human	CCGGAGAAGACTTGGCCTCTAATTTCTCGAGAAATTAGAGGCCAAGTCTTCTTTTTTG
MLKL #1	mouse	CCGGAGATCCAGTTCAACGATATATCTCGAGATATATCGTTGAACTGGATCTTTTTTG
MLKL #2	mouse	CCGGTCCCAACATCTTGCGTATATTCTCGAGAATATACGCAAGATGTTGGGATTTTTG
p65 #1	mouse	CCGGGGAGTACCCTGAAGCTATAACCTCGAGGTTATAGCTTCAGGGTACTCCTTTTTG
p65 #2	mouse	CCGGAGGCCATATAGCCTTACTATCCTCGAGGATAGTAAGGCTATATGGCCTTTTTTG

Supplementary Table 2. Primers used in this study.

Gene	Species	Primer sequence (5’-3’)
Cxcl8	human	Forward: CAGTTTTGCCAAGGAGTGCT; Reverse: ACTTCTCCACAACCCTCTGC
Cxcl1	human	Forward: CCCCAAGAACATCCAAAGTGT; Reverse: TGGATTTGTCACTGTTCAGCA
Cxcl2	human	Forward: CACTCAAGAATGGGCAGAAAG; Reverse: TCAGGAACAGCCACCAATAAG
Csf1	human	Forward: TGGCGAGCAGGAGTATCAC; Reverse: AGGTCTCCATCTGACTGTCAAT
Iκbα	human	Forward: GATCCGCCAGGTGAAGGG; Reverse: GCAATTTCTGGCTGGTTGG
Tnfα	human	Forward: CCCAGGCAGTCAGATCATCT; Reverse: GGACCTGGGAGTAGATGAGG
Relb	human	Forward: TTTTAACAACCTGGGCATCC; Reverse: CGCAGCTCTGATGTGTTTGT
β-Trcp2	human	Forward: AAACCAGCCTGGAATGTTTG; Reverse: CAGTCCATTGCTGAAGCGTA
Gapdh	human	Forward: TTGCCATCAATGACCCCTTCA; Reverse: CGCCCCACTTGATTTTGGA
Cxcl1	mouse	Forward: CTATCGCCAATGAGCTGCG; Reverse: TCTGAACCAAGGGAGCTTCA
Cxcl2	mouse	Forward: CTCTCAAGGGCGGTCAAAAAGTT; Reverse: TCAGACAGCGAGGCACATCAGGTA
Cxcl3	mouse	Forward: AGACCATCCAGAGCTTGACG; Reverse: GGACTTGCCGCTCTTCAGTA
Cxcl5	mouse	Forward: TTCCTCAGTCATAGCCGCAA; Reverse: TGGATCCAGACAGACCTCCT
Cxcl7	mouse	Forward: TCAGACCTACATCGTCCTGC; Reverse: AGTGAACTCCTGGCCTGTAC
Csf1	mouse	Forward: ATGGACACCTGAAGGTCCTG; Reverse: GTTAGCATTGGGGGTGTTGT
Csf2	mouse	Forward: TTGGAAGCATGTAGAGGCCA; Reverse: CGCCCTTGAGTTTGGTGAAA
Il6	mouse	Forward: TACCACTCCCAACAGACCTG; Reverse: GGTACTCCAGAAGACCAGAGG
Il1b	mouse	Forward: ACTCATTGTGGCTGTGGAGA; Reverse: TTGTTCATCTCGGAGCCTGT
Ifng	mouse	Forward: TTCTTCAGCAACAGCAAGGC; Reverse: ACTCCTTTTCCGCTTCCTGA
Iκbα	mouse	Forward: TGACTTTGGGTGCTGATGTC; Reverse: AAGCTGGTAGGGGGAGTAGC
β-Actin	mouse	Forward: CAGCCTTCCTTCTTGGGTAT; Reverse: GGTCTTTACGGATGTCAACG
